# Comparing objective measures of environmental supports for pedestrian travel in adults

**DOI:** 10.1186/1476-072X-8-62

**Published:** 2009-11-19

**Authors:** Elizabeth Shay, Daniel A Rodriguez, Gihyoug Cho, Kelly J Clifton, Kelly R Evenson

**Affiliations:** 1 Institute for the Environment, University of North Carolina Chapel Hill, Chapel Hill NC, USA; 2 Department of City and Regional Planning, University of North Carolina, Chapel Hill NC, USA; 3 National Center for Smart Growth, University of Maryland, College Park MD, USA; 4 Department of Epidemiology, Gillings School of Global Public Health, University of North Carolina, Chapel Hill NC, USA

## Abstract

**Background:**

Evidence is growing that the built environment has the potential to influence walking--both positively and negatively. However, uncertainty remains on the best approaches to representing the pedestrian environment in order to discern associations between walking and the environment. Research into the relationship between environment and walking is complex; challenges include choice of measures (objective and subjective), quality and availability of data, and methods for managing quantitative data through aggregation and weighting. In particular, little research has examined how to aggregate built environment data to best represent the neighborhood environments expected to influence residents' behavior. This study examined associations between walking and local pedestrian supports (as measured with an environmental audit), comparing the results of models using three different methods to aggregate and weight pedestrian features.

**Methods:**

Using data collected in 2005-2006 for a sample of 251 adult residents of Montgomery County, MD, we examined associations between pedestrian facilities and walking behaviors (pedestrian trips and average daily steps). Adjusted negative binomial and ordinary least-squares regression models were used to compare three different data aggregation techniques (raw averages, length weighting, distance weighting) for measures of pedestrian facilities that included presence, condition, width and connectivity of sidewalks, and presence of crossing aids and crosswalks.

**Results:**

Participants averaged 8.9 walk trips during the week; daily step counts averaged 7042. The three aggregation techniques revealed different associations between walk trips and the various pedestrian facilities. Crossing aids and good sidewalk conditions were associated with walk trips more than were other pedestrian facilities, while sidewalk facilities and features showed associations with steps not observed for crossing aids and crosswalks.

**Conclusion:**

Among three methods of aggregation examined, the method that accounted for distance from participant's home to the pedestrian facility (distance weighting) is promising; at the same time, it requires the most time and effort to calculate. This finding is consistent with the behavioral assumption that travelers may respond to environmental features closer to their residence more strongly than to more distant environmental qualities.

## Background

Consensus is growing that the built environment has the potential to influence walking--both positively and negatively. Because pedestrian-supportive features of the built environment often are expensive and relatively permanent, and thus should be designed and placed carefully, it is important to understand the nuances of which facilities in which contexts are associated with changes in walking behavior. This understanding can increase the likelihood that environmental design will produce desirable effects among travelers. Extensive research has looked at the level of active travel (travel to and from destinations by physically active modes such as walking or bicycling) as it relates to environments characterized as dense, well-connected, safe, and diverse in terms of land use.

Despite emerging research, relatively little is known about how the pedestrian infrastructure, including sidewalks and crossing aids as well as other pedestrian-supportive design features, at the very local scale relates to active travel. A focus on those infrastructure supports that can help pedestrians negotiate street traffic is important because perceived safety has been associated with higher incidence of walking and bicycling to school [1,2]. With few exceptions [3], high vehicle traffic has been associated with lower levels of physical activity in nearby areas [4,5].

Saelens and Handy [6] summarize current knowledge about environmental correlates of walking, and show that commonly accepted pedestrian infrastructure supports (e.g., sidewalk quality, connectivity) have positive, negative or no associations with walking. For some attributes, such as sidewalk access [7,8] and street connectivity [9], evidence about their association with walking is emerging. Frank et al. [10] combined land use mix, residential density and street connectivity into a walkability index, and found the index to correlate with objectively measured physical activity. Hoehner et al. [11] found associations between active travel and sidewalk characteristics. With few exceptions [12], individual micro-environmental features, such as sidewalk width and crossing aids, remain largely unexplored.

It is possible that measurement techniques themselves obscure important relationships that could inform planning decisions. Even detailed and localized measures of physical features must make accommodations in selecting a geographic scale and in drawing boundaries. Nelson et al. [13] call for more nuanced representation of residential landscapes, as the simple urban/suburban/rural designation may obscure patterns in physical activity in various environments. Connectivity and access may be underestimated if the local pedestrian environment is not accounted for *beyond *the road network [14] (i.e., including the off-road pedestrian environment). The inconsistent findings and mixed results to date in studies examining walking behavior and pedestrian supports [15,16] may reflect weak measures of the environment.

The problems are several. First, the majority of studies focusing on pedestrian supports use self-reported data [4,15-19]. This kind of data has been inconsistently associated with objective data [20-22]. Yet, objective measures display associations with physical activity that are independent of perceived or self-reported measures, suggesting that using both may yield a better understanding of the interplay of environment and physical activity [21,23].

Second, data on sidewalks, paths, and other pedestrian facilities may be of uncertain quality; some sources may not distinguish between missing data and absent pedestrian facilities. Studies that examine very local conditions and contexts may be limited by the type and volume of data that municipal, county, or other sources can provide. Finally, studies that aggregate pedestrian environmental features into areas or neighborhoods surrounding each study participant often assign the same weight to features regardless of location relative to individuals' residences; this fails to account for the behaviorally relevant influence of the environment closer to home, and raises boundary issues, with most studies to date treating the area within a circumscribed environment uniformly.

In this study, we examine associations between walking and street-level pedestrian supports. In light of the challenges (described above) of representing the pedestrian environment, including self-reported physical activity and environmental features and uneven data sources, this study uses accelerometers along with reliable and objective environmental audit data. The study also addresses the challenge of scale and uniformity of geographical units by comparing traditional measures with measures that account for road segment length and distance between the residence and pedestrian features, and discussing how the use of these various measures produces different results in models of walking behavior.

## Methods

### Study Sample

We employ built environment and physical activity measures, along with self-reported personal characteristics and travel activity, in behavioral models (walk trips and steps) using three different data aggregation techniques. In addition to the raw average, we weight by length to capture the impact of longer segments and the attendant greater number of facilities that may encourage walking, and by distance to account for the possibility that a remote pedestrian facility poses a barrier to walking behavior while a nearby facility may encourage walking.

This study is based on data collected in Montgomery County, Maryland--a region with a variety of built environments, ranging from low-density residential-only to dense, transit-oriented areas with mixed land uses. The American Community Survey http://www.census.gov/acs/www/ reports a 3-year (2005-0707) average estimate of 917,181 residents of Montgomery County (excluding group housing) with a median household income of $89,284 and average travel time to work of 33 min.

**Figure 1:**
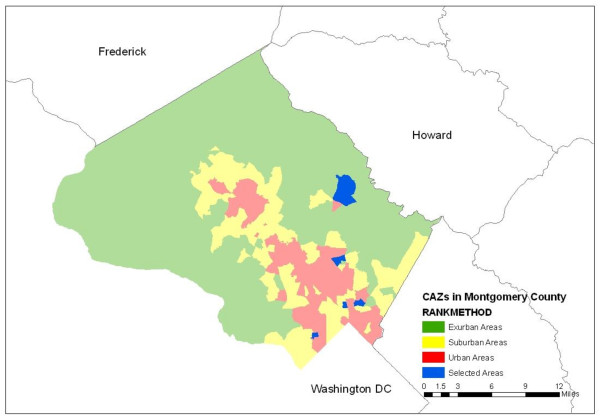
**Five areas selected for audit and travel survey (blue): two urban, two suburban, one exurban**.

To identify walkable areas in Montgomery County, each of the 318 zones used by county staff for community planning were characterized according to their development characteristics (density of population, employment, open space and housing), motorized activity (proximity to bus and rail, percentage of population taking transit to work in 2000, and roadway and bus route density), and pedestrian infrastructure (sidewalk connectivity, sidewalk coverage, and percentage of population walking or cycling to work in 2000). A built environment score [24] then was used as a basis to classify zones into one of three types of built environments using factor and cluster analysis: high walkability (urban, 30 zones), moderate walkability (suburban, 135 zones) and low walkability (exurban, 153 zones). Two zones were selected at random from the urban group, two from the suburban group, and one from the exurban group. Only one low-walkability zone was included because such zones by definition cover a larger land area (average 1720 acres) relative to urban (average 167 acres) and suburban areas (average 415 acres), thus requiring substantially greater resources for field data collection. Figure 1 shows the study areas.

Participants were recruited from the five zones with several methods, including invitations mailed to neighborhood residents, telephone calls to matched residents in the study area, door-to-door recruitment, and mass media coverage in local newscasts and newspapers. A total of 293 participants were enrolled between April 2004 and September 2006 as part of a convenience sample from the five zones, including 47 from Bethesda (urban), 44 from Forest Glen (urban), 67 from Four Corners (suburban), 62 from Layhill (suburban), and 73 from Olney (exurban). The percentage of housing units in each zone represented in the sample range from 3.0% (Bethesda) to 7.3% (Four Corners). Participants were healthy, able-bodied adults who resided in one of the five zones and were not absent from the metropolitan area during the study period. Each of the participants completed an in-person computer-assisted survey; they also underwent anthropometric measurements, wore an accelerometer for seven days, and concurrently completed a travel diary. The study was approved by the Institutional Review Board, and all participants gave written informed consent.

### Walking Outcomes

Dependent variables included two different outcome measures: number of walk trips during the study week and average daily steps. The travel diary completed by each participant reported the number of walk trips. For each trip, participants were instructed to enter the departure time, travel mode (auto, rail, bus, walk/run, bicycle or other), arrival time and arrival location. A pilot study using portable GPS (global positioning system) units concurrently with the diary showed moderate to high agreement for the number of walk trips taken during the week (Cho GH, personal communication).

Objective data on steps were collected from accelerometers (ActiGraph model 7164; Pensacola FL) worn by the participants for the seven days of travel recorded for the study. Le Masurier and Tudor-Locke [25] found acceptable performance of the step count function using this accelerometer, with no differences between actual steps and accelerometer-determined steps except at slow walking speeds. The small lightweight unit, worn on the hip on either a belt clip or in a nylon pouch and thus unlikely to interrupt normal physical activity, was set to measure both activity counts and steps in 1-minute epochs. Participants were instructed to wear the unit while awake, and to remove it only when sleeping or exposed to water (bathing or swimming). A day was considered valid if the participant wore the unit for at least 10 hours. Having a minimum of two valid days qualified a participant for inclusion in the data analysis. The average number of daily steps was calculated by totaling daily values for valid days and dividing it by the total number of valid days.

### Neighborhood Characteristics

Unlike the majority of previous research that relies on secondary data to characterize the built environment, we conducted a street-by-street walking audit using the Pedestrian Environment Data Scan (PEDS) for all street segments (n = 3636) within 1/2 mile (800 m) of where participants lived in each of the five zones. The PEDS method is intended as an efficient and reliable instrument to capture a range of elements of the built and natural environment, integrating handheld technology with visual assessment of physical features. PEDS was developed from the SPACES (Systematic Pedestrian and Cycling Environment Scan) audit [26] and adjusted for conditions in the United States; the PEDS method includes extensive supporting documentation and training materials. It has been shown to have adequate test-retest reliability in the context of the study areas in question [27]. Street segments were defined as the road or pedestrian path bounded by cross-streets or intersections. When segments were longer than 700 ft, the segment was subdivided to ensure consistency in the comparison across segments. Two-person teams covered the street segments in each zone, where they traversed path and sidewalk centerlines and noted and evaluated pedestrian features.

Each street segment was measured for attributes commonly unavailable from secondary data, such as the presence, quality and width of pedestrian facilities, characteristics of adjacent roads, the presence of pedestrian supports (including road crossing aids), and the design characteristics of the built environment along the segments. Our measures include the presence of sidewalks or trails (percentage of links in a participant's buffer where at least one of these features is present); sidewalk condition (good/not good, determined visually with a descriptive quality assessment rubric); connections (number of connections to other sidewalks and paths); sidewalk or trail width over 4' (1.22 meters); presence of crossing aids (e.g., stop lights, stop signs, pedestrian island, and pedestrian-supportive signage to alert motorists to watch for or yield to walkers); and presence of crosswalks.

Although street-segment audits provide rich environmental information, the data must be aggregated to usefully characterize the local environment of participants. Neighborhoods were person-specific, defined as the area covered by circles of 1/2-mile radius drawn around each person's home location--an appropriate scale for considering the micro-environments that may be important for walking [11]. We employed and then tested three ways of aggregating the audit data of interest. First, we used simple averages, expressed as the percentage of audited segments that include a given attribute. This first measure may fail to account for the influence of different road segment lengths, or distance from residences to the segments. Second, we weighted each segment by its length, yielding the percentage of road length that contains the same attribute. Weighting by length may capture the impact of longer segments and the attendant greater presence of facilities that may affect the likelihood of residents choosing to walk. Finally, we weighted each segment by the inverse distance from the segment's midpoint to the participant's home. Thus, closer segments were weighted more heavily than more remote segments, reflecting our expectations that the influence of an environmental feature may be stronger when located closer to a participant's residence.

Formally, for each participant *j*, the first measure was calculated as:

*V*_*ij *_is the value of a variable for segment *i*, located within the neighborhood of participant *j *and *n *is the number of segments within the neighborhood of participant *j*. The second measure was calculated as:

*l*_*ij *_is the length of segment *i*, also within the neighborhood of participant *j *and all other variables are as defined before. The third measure was:

*d*_*ij *_where is the distance from the midpoint of segment *i *to the participant's *j *home location and all other variables are as defined before.

Given the acknowledged relevance of density for transport-related physical activity, we included as a covariate each neighborhood's population density (hundreds of persons per acre), measured using block-level population data from the 2000 U.S. Census and dividing it by the land area in parcels. When a block was not fully contained within a neighborhood, its population was assigned in direct proportion to the area of the block contained within the neighborhood, which assumes a uniform population density within each block. All measures were derived using ArcGIS 9.2 (Environmental Systems Research Institute Inc., Redlands CA, 2006).

### Individual Characteristics

Self-reported socio-demographic measures collected with the survey include age, sex, and education. Age was included as a continuous integer variable (after testing a squared age term to check for linearity), while sex (female = 1) and education (college degree = 1) were included as binary variables.

### Statistical Analyses

Of the 262 participants for whom we have complete data on steps, seven fell short of the threshold of two valid days of accelerometer data. Another was eliminated because of missing values for a control variable. Three others were identified as having outlying step counts (more than seven standard deviations away from the mean, likely the result of faulty accelerometers). The final sample comprised the remaining 251 participants.

For the outcome variable of number of walk trips, we used count regression models, to avoid the inefficient and biased estimates that may result from applying ordinary least-squares regression. Unlike Poisson, negative binomial models do not assume equivalence of the dependent variable's mean and variance. For the outcome variable of average daily steps, we used ordinary least-squares regression. In light of evidence of over-counting of steps during slow walking by the accelerometer in use [25], we followed the advice of Tudor-Locket et al. [28] and censored the step counts associated with low activity. As a result, any steps registered when accelerometer counts were below 500 per minute were set to zero. As a reference, a study of slow walking (3 kph) in free-living conditions showed that 95% of all observations registered with accelerometers were greater than 972 counts/minute [29].

The six pedestrian facilities that served as exposure (independent) variables were converted from continuous variables into tertiles (thirds) to identify potential dose-response relationships and facilitate discussion. Separate models were examined for each pedestrian facility, using the middle and top tertiles as independent variables and the lower tertile as the reference category, while controlling for population density and individual characteristics. For each facility, we separately modeled the three different aggregation techniques: averages, length weighting, and distance weighting. Correlations among the techniques are described in the results.

In addition to a model for each exposure (a pedestrian facility), we created an index of facilities by summing the tertile score (0, 1 or 2) for each pedestrian facility across the aggregation method. For example, if an observation fell in the top tertile for all six pedestrian facilities, it would have an index score of 12; if it was in the lowest tertile across all six facilities, it would have an index score of 0. Scores were calculated for all three aggregation techniques. We created the scores rather than pooling the data (that is, using all six pedestrian facility variables in a single model) because of the high collinearity among the pedestrian facilities; variance inflation factors for the six facilities ranged from 3.0 (sidewalk condition) to 8.5 (crosswalks). These indices were tested for linearity with models that included the squared index value, with no meaningful difference in the results.

To compare the models using the indices for the three different aggregation methods, we use the Bayesian Information Criterion prime (BIC'), which reflects the relative performance of models. Raftery [30] suggests that the evidence favoring one model over another is weak, positive, strong, or very strong if the absolute difference in BIC' (or the related BIC) for two models is 0-2, 2-6, 6-10, or >10, respectively. All statistical analyses were performed in Stata 10.1 (College Station, TX).

## Results

Table 1 describes the participants in our sample and their neighborhood built environment. Study participants ranged in age from 19 to 90 years, with a mean of 50. Two-thirds (66%) were female; and 84% had a college degree (much higher than the 57% reported for Montgomery County residents in the 2005-2007 American Community Survey). The self-reported number of walk trips during the travel week averaged nearly 9, ranging from 0 to 43 trips. Walking, measured objectively with the pedometer function of the accelerometer, averaged 7042 censored steps per day.

**Table 1:** Descriptive statistics for participants, including socio-demographic, pedestrian environment, and physical activity measures (n = 251)

Variable		Mean	SD	Minimum	Maximum	Range of middle tertile	
**Socio-demographic measures**							
Age, continuous		50.49	14.29	19	90		
Female (binary; 1 = yes)		.66	.47	0	1		
College (binary; 1 = college degree)		.84	.37	0	1		
**Physical activity**							
Walk trips (number in week)		8.86	9.22	0	43		
Steps (average censored daily)		7042	3173	0	18046		
							
**Pedestrian environment**	**Aggregation technique**						
Presence of sidewalks	average	.59	.16	.05	.92	.50	.63
	length	.59	.17	.06	.93	.49	.64
	distance	.60	.20	.05	1.18	.50	.67
Good sidewalk condition	average	.78	.16	.14	1.00	.70	.91
	length	.76	.17	.04	1.00	.69	.88
	distance	.77	.25	.08	1.95	.70	.88
Connections	average	3.30	.47	2.00	4.22	2.99	3.58
	length	3.35	.47	2.00	4.36	3.06	3.63
	distance	3.31	.93	1.66	7.23	2.78	3.59
Wide sidewalks	average	.96	.03	.86	1.00	.96	.98
	length	.94	.04	.83	1.00	.93	.97
	distance	.99	.21	.53	2.15	.89	1.03
Crossing aid present	average	.30	.15	.00	.61	.24	.33
	length	.31	.15	.00	.62	.26	.39
	distance	.29	.23	.00	1.32	.18	.27
Crosswalk present	average	.27	.14	.00	.68	.21	.32
	length	.28	.14	.00	.67	.22	.34
	distance	.25	.20	.00	1.19	.16	.25

Table 1 also shows values for the six pedestrian features, each measured in three different ways: raw average for the buffer, weighted by length, and weighted by distance. In the far right columns are the range of values for the middle tertile, which also serve as the upper bound on the lowest tertile and lower bound on the highest tertile; that is, for each variable, one-third of measures fell below the lower value in range, and one-third above the upper value. Although the three different values are of the same order of magnitude for a given pedestrian facility, comparisons across the three ways of summarizing the neighborhood's environmental variables are limited because each method (average, length-based and distance-based) is constructed differently. Pearson correlations across aggregation techniques tended to be high. The raw averages measure and the length-weighted measure had the highest correlation (range 0.89-0.99 for sidewalk width and condition, respectively), while correlations between length-weighted and distance-weighted measures were lowest (range 0.23-0.79 for sidewalk width and presence, respectively).

Table 2 shows the results of adjusted associations between the number of walk trips taken in the week and individual pedestrian facilities. Among the average measures, sidewalk condition was positively associated with weekly trips (for the middle tertile), with an incidence rate ratio (IRR) suggesting nearly twice as many (1.85 times) trips for those with sidewalk conditions scoring in the middle tertile than those in the low (reference) tertile. Sidewalk condition for the highest tertile was not significant. The high tertile for presence of crossing aids is significant, with an IRR indicating 48% more weekly trips associated with this pedestrian facility. Against our expectations, for wide sidewalks, the highest tertile is statistically but negatively associated with walk trips, with an IRR of .68, suggesting 32% fewer walk trips for the high category.

**Table 2:** Adjusted IRR^1 ^with 95% CI^2^, association between weekly walk trips and individual pedestrian facilities (n = 251)

	Average			Weighted by length			Weighted by distance		
	
	IRR	(95% CI)	**BIC'**^**3**^	IRR	(95% CI)	BIC'	IRR	(95% CI)	BIC'
Sidewalks present			9.71			10.58			10.60
Medium	1.11	(.78, 1.59)		1.06	(.74, 1.52)		.98	(.68, 1.40)	
High	.94	(.66, 1.34)		1.06	(.74, 1.51)		.94	(.65, 1.35)	
									
Sidewalk condition			-3.29			0.34			3.88
Medium	1.85	(1.30, 2.62)***		1.81	(1.26, 2.61)***		1.25	(.89, 1.75)	
High	1.19	(.80, 1.77)		1.36	(.94, 1.96)		1.61	(1.13, 2.29)***	
									
Sidewalk connections			7.11			4.34			9.75
Medium	1.08	(.77, 1.51)		1.01	(.73, 1.42)		.86	(.61, 1.22)	
High	.78	(.55, 1.10)		.68	(.48, .96)**		1.00	(.70, 1.42)	
									
Sidewalk width>4'			1.05			8.32			8.79
Medium	1.15	(.80, 1.64)		1.12	(.81, 1.55)		.95	(.68, 1.34)	
High	.68	(.49, .94)**		.84	(.59, 1.20)		1.21	(.85, 1.73)	
									
Crossing aid present			5.84			4.92			4.47
Medium	1.12	(.79, 1.59)		.97	(.70, 1.35)		1.26	(.90, 1.78)	
High	1.48	(1.03, 2.12)**		1.43	(1.01, 2.03)**		1.63	(1.11, 2.37)**	
									
Crosswalk			9.92			6.11			7.91
Medium	1.15	(.80, 1.65)		1.03	(.72, 1.45)		1.33	(.94, 1.88)	
High	1.17	(.80, 1.72)		1.40	(.98, 1.99)		1.29	(.86, 1.92)	

All models control for: age, sex, education, and population density;

**significant at p < 0.05; ***significant at p < 0.01

The lowest tertile is the reference category for each pedestrian facility.

^1^Incidence rate ratio

^2 ^Confidence interval

^3^Bayesian Information Criterion

Using the length-weighted measure for the same dependent variables, the results are similar to the average measures for sidewalks in good condition (middle tertile) and crossing aids (high tertile). However, sidewalk width was no longer significant, while sidewalk connections show a negative association of the high tertile with weekly walk trips.

For the distance-weighted measures, sidewalk condition was positively associated with walking, but in this case for the high category. Sidewalk connections and width were not significant. Finally, crossing aids (high tertile) were positively associated with walk trips, with an IRR suggesting 63 percent more walk trips for the high tertile compared to the low, all else held equal.

Across the three methods, various aggregations reveal somewhat similar associations of trips with pedestrian facilities. For the facilities that show statistically significant associations for all aggregations (sidewalk condition and crossing aids), the BIC' values provide positive evidence that the average measure for sidewalk condition is favored over the length weighting, and strong evidence favoring average over distance weighting. By contrast, the BIC' reveals weak evidence favoring the distance weighting over both the length weighting and average for the presence of crossing aids.

Table 3 shows the results of combining the pedestrian facility scores into indices and using them as single independent variables (one for each of the three aggregation methods), along with the same four controls. Education and population density were positively significant for all three aggregation techniques. None of the indices were significant at the 95% confidence level; however, the distance-weighted index has a p-value of 0.082 (p values are 0.806 and 0.366 for average and length weighting, respectively). The BIC' values provide positive evidence favoring the distance weighting technique over both the other two aggregation methods.

**Table 3:** Adjusted IRR^1 ^with 95% CI^2^, association between weekly walk trips and index of pedestrian facilities (n = 251)

	Average		Weighted by length		Weighted by distance	
	
	IRR	(95% CI)	IRR	(95% CI)	IRR	(95% CI)
Age	.99	(.98, 1.00)	.99	(.98, 1.00)	.99	(.98, 1.00)
Female	.80	(.59, 1.07)	.80	(.60, 1.08)	.82	(.62, 1.12)
College	1.65	(1.11, 2.44)**	1.63	(1.10, 2.40)**	1.62	(1.10, 2.40)**
Density	1.07	(1.02, 1.12)***	1.06	(1.02, 1.10)***	1.05	(1.02, 1.09)**
Index of ped facilities	.99	(.93, 1.06)	1.03	(.97, 1.09)	1.04	(.99, 1.09)^4^
**Summary statistics**						
LR statistic		-798.80981		-798.43181		-797.34803
P(alpha)		0.0004		0.0003		0.0001
Pseudo-R^2^		0.0139		0.0144		0.0157
BIC'^3^		5.13		4.37		2.20

**significant at p < 0.05; ***significant at p < 0.01

^1^Incidence rate ratio

^2 ^Confidence interval

^3^Bayesian Information Criterion

^4^p = 0.082

Table 4 presents models for censored steps regressed against each individual pedestrian facility and the control variables. For the average measures, only sidewalk condition was significant (for the high tertile), with an unexpected negative coefficient; no other average measures of pedestrian facilities were significant for steps. For length weighting, presence and connectivity of sidewalks was significant, with a coefficient suggesting an additional 1142 steps for participants residing in locations scoring in the top tertile for presence of sidewalks and 995 more steps for the middle tertile for sidewalk connections. Wide sidewalks also showed a positive association with steps for the middle tertile of the length weighting, with 914 steps.

**Table 4:** Adjusted OLS^1^, number of average daily censored steps against individual pedestrian facilities with 95% CI^2 ^(n = 251)

	Average			Weighted by length			Weighted by distance		
	
	Coefficient	(95%CI)	**BIC'**^**3**^	Coefficient	(95% CI)	BIC'	Coefficient	(95% CI)	BIC'
Sidewalks present			-1.60			-3.57			-5.48
Medium	403	(-580, 1387)		546	(-419, 1511)		850	(-92, 1792)	
High	921	(-10, 1853)		1142	(208, 2077) **		1312	(376, 2248)***	
									
Sidewalk condition			-10.18			-6.26			2.00
Medium	368	(-577, 1313)		331	(-643, 1306)		45	(-887, 978)	
High	-1254	(-2276, -231)**		-984	(-1978, 9)		276	(-705, 1256)	
									
Sidewalk connections			-1.66			-2.55			-8.40
Medium	925	(-5, 1854)		995	(80, 1909)**		-21	(-928, 885)	
High	571	(-346, 1488)		300	(-616, 1217)		1304	(381, 2227)***	
									
Sidewalk width>4'			-0.02			-1.83			-6.85
Medium	292	(-685, 1270)		914	(22, 1806)**		668	(-232, 1569)	
High	-434	(-1331, 464)		362	(-592, 1316)		1458	(507, 2409)***	
									
Crossing aid present			1.45			-0.40			-0.40
Medium	-337	(-1287, 614)		-686	(-1595, 224)		-702	(-1662, 257)	
High	78	(-922, 1077)		-51	(-1026, 924)		-92	(-1161, 977)	
									
Crosswalk			2.21			1.00			1.57
Medium	-155	(-1160, 849)		341	(-621, 1302)		31	(-933, 995)	
High	8	(-1022, 1037)		567	(-403, 1536)		424	(-650, 1498)	

All models control for: age, sex, education, and population density

**significant at p < 0.05; ***significant at p < 0.01

The lowest tertile is the reference category for each pedestrian facility.

^1^OLS: ordinary least-squares regression

^2^Confidence interval

^3^Bayesian Information Criterion

For distance weighting, we found an additional 1312 steps for those in the high tertile for presence of sidewalks, and 1304 steps for the high tertile for the sidewalk connections. Wide sidewalks showed a positive association with the high tertile of the distance weighting, with 1458 steps. For the three pedestrian facilities with statistically significant associations with steps for length and distance weighting, the BIC' values were positive evidence favoring the distance weighting over length. The crossing aids and crosswalks were not associated with steps for any of the aggregation techniques.

We reexamined the models by not censoring steps associated with low activity (fewer than 500 counts/minute), and compared the results. We elected to retain the censored step models, because they revealed additional associations with pedestrian facilities (compared with uncensored) and because the lower BIC' values favor the censored models. We found the results for sidewalk condition and width and presence of crossing aids to be generally the same for censored and uncensored, while sidewalk presence and connections were not significant for the uncensored steps. One pedestrian facility was significantly associated with steps for uncensored steps but not for censored--the presence of crosswalks for the high tertile with distance weighting.

In addition, we ran models that included a variable for average wear time (total wear time on valid days divided by number of valid days) to control for undue influence of participants with longer recording periods, and found no substantive differences in the results, in terms of significance, sign, or magnitude of association. These results are as expected, since valid days already are defined to be long--those on which the accelerometers were worn for more than 10 hours. This also applies to the models using the index of pedestrian facilities (Table 5, below).

**Table 5:** Adjusted OLS^1^, number of average daily censored steps against index of pedestrian facilities with 95% CI^2 ^(n = 251)

	Average		Weighted by length		Weighted by distance	
	
	Coefficient	(95% CI)	Coefficient	(95% CI)	Coefficient	(95% CI)
Age	-71	(-97, -44)***	-71	(-98, -45)***	-72	(-98, -46)***
Female	-950	(-1759, -141)**	-937	(-1744, -130)**	-905	(-1703, -107)**
College	134	(-907, 1175)	143	(-895, 1181)	95	(-932, 1121)
Density	-11	(-124, 101)	-32	(-78, 270)	-82	(-193, 28)
Index of pedestrian facilities	9	(-185, 203)	96	(-78, 270)	174	(44, 303)***
**Summary statistics**						
F statistic		6.40		6.66		7.99
R^2^		0.1155		0.1197		0.1401
Adjusted R^2^		0.0974		0.1017		0.1226
BIC'^3^		-3.17		-4.37		-10.27

**significant at p < 0.05; ***significant at p < 0.01

^1^OLS: ordinary least-squares regression

^2^Confidence interval

^3^Bayesian Information Criterion

For the step models using the index (Table 5), only the distance weighting showed a statistically significant association with steps. The BIC' values provided strong positive evidence of distance weighting being preferred over both the length-weighting and averages, with the former weakly favored over the latter. Age and sex (female) were significantly and negatively associated with censored steps in all three models. The same models run without censoring of steps were less informative, with BIC' values suggesting the censored models are preferable.

## Discussion

This study draws on objective measures of the built environment and physical activity, along with self-reported socio-demographic characteristics and travel activity, to examine data aggregation techniques and their impact on associations with walking behavior. Using data from travel diaries and an environmental audit of five neighborhoods in Montgomery Co. (Maryland), this study considered three different techniques for aggregating quantitative measures of pedestrian facilities on road segments, starting with a 1/2-mile radius (800 m) buffer around each study participant. A raw average, while the simplest and easiest to calculate, may fail to account for the influence of different road segment lengths, or distance from residences to the segments. A length weighting considers the possibility that longer segments provide more facilities that may affect the likelihood of residents choosing to walk. By contrast, weighting by distance makes the behavioral assumption that a pedestrian facility that is remote and more difficult to access poses a barrier to walking behavior, while a nearby facility may encourage walking.

Comparing the three aggregation methods within sets of models, the length-weighted measures generally were similar to the average measures in significance as well as magnitude and direction of associations. Specifically, trips were higher in the presence of good sidewalk condition and crossing aids. The distance-weighted measures showed a positive association between trips with sidewalk condition and crossing aids and crosswalks, with a larger coefficient than either of the other two aggregation techniques.

Overall, there were fewer relationships observed in the step models using average measures, consistent with some earlier observations [31] that number of trips appears to have a stronger relationship with certain environmental features than do physical activity measures. For example, Rutt and Coleman [32] found no link between walking duration and environmental measures. There is little research available on step counts and environmental qualities. However, our length- and distance-weighted models revealed more associations between facilities and steps than did the average measures. Moreover, our models using censored steps (eliminating those associated with low activity) found more associations with pedestrian facilities than we observed in models that included all steps.

For step models, the length weighting was not as similar to the averages as with trip models; for some measures it produced results more comparable to the distance weighting, which BIC' values suggest are preferable. Additional studies are needed to confirm the findings in this single small study. Given the time and effort required to produce a length-weighted measure and the limited variation in results when compared to average measures for the same models, this aggregation method may not provide much marginal benefit in policy-relevant findings and may not be worth pursuing in the absence of a research question for which link length is important. The distance weighting goes beyond accounting for spatial attributes of the road segments within a buffer zone to make a behavioral assumption; specifically, greater distance to road segments and access to the associated pedestrian features may inhibit walk trips, while closer proximity to such physical features may encourage walking. In our study, the distance-weighted measures produced apparently more discerning results with better model fit than the average measures, and may be worth developing if resources permit.

Comparing the results of two sets of models, for trips and steps, revealed that higher numbers of trips were associated with sidewalks in good condition and with crossing aids. The latter are of particular interest, since they were found to be significant in models with all three aggregations. The crossing aids that were recorded in the audit include pedestrian-related traffic signs such as "yield to," "watch for," or "share the road with...," as well as flashing warnings or hard structures such as traffic islands and overpasses. These crossing aids represent potential tools that, while not trivial in their need for careful planning and placement, are relatively inexpensive and flexible in time and space, compared to longer-term and more ambitious strategies such as road design or development and building regulations.

The negative association of sidewalk connections and trips (revealed by the length weighting), although surprising, is not entirely unprecedented; Clifton and Dill [33] found the same negative association between street connections in a 1/2-mile buffer and trip frequencies. It is possible that our length-weighting measure may have introduced bias into the analysis of certain environmental features like the presence of crossing aids, crosswalks, and sidewalk connections. When weighting by length, a short segment would be disfavored relative to a long segment (i.e., a short segment with a crosswalk is better than a long segment with one); by contrast, for an attribute present throughout the segment (like sidewalks), weighting by length would favor longer segments to shorter ones. Although the variation in segment length was fairly small in our sample, weighing by length suggests that longer segments with such features are negatively associated with walking trips, relative to shorter segments. By contrast, weighting by length is desirable when the attribute is present along the entire segment, as with the sidewalks variable. We therefore suggest that segment length-based weights should be used with caution.

The models for steps showed more sensitivity to sidewalks--both their presence and their attributes--than to crossing aids and crosswalks, while the trip models showed more associations with crossing aids and fewer or more divergent results with sidewalk qualities. Given that walk trips--and the steps that constitute them--respond to crossing aids and sidewalk condition, it would be interesting to consider, in future work, how trips for different purposes (shopping, exercise, sociability, etc.) vary, in light of known differences in sensitivity to pedestrian facilities depending on trip type [34].

The use of objective street-level information is a unique strength of this study. Objective data provide valuable checks on subjective data, and lets researchers compare perceived with measured conditions and associated behavior. Although quantitative, objective measures of the environment have proliferated as tools for manipulating and presenting data have become more sophisticated, problems remain in developing standard definitions and valid measures [35]. Of the three aggregation measures examined, the measure that accounts for distance from participant's home to pedestrian facilities was most informative, and conformed with behavioral expectations that the environment closer to one's residence should exert a stronger influence than that further away. At the same time, this measure came at a cost in terms of time and effort required to calculate it. The length weighting differs only marginally from the average measure, and may not merit the time and resources required to calculate it, at least for these specific pedestrian facilities.

Although the length- and distance-weighted measures offer interesting refinements to the raw averages, there are substantial limitations and uncertainties. We do not know whether the effect of the built environment on physical activity has a linear or inverse association with distance from one's residence. This assumption is particularly problematic when length and distance are small numbers. For example, the effect of a facility that is 100' from a residence might be twice as great as one 200' away; alternatively, there may be a threshold effect, beyond which distance doesn't matter. Additional examination and refinement of aggregation techniques and prospective study design are in order. In addition, it would be worthwhile to examine differences among functional types of streets (e.g., arterial vs. local) where traffic conditions and threats to safety vary. Several other data breakdowns provide rich possibilities for future investigation into how the various aggregation techniques differ in useful interpretable and policy-relevant results. Specifically, trips for various purposes (with trips ends characterized as home, work, and other types of destinations), and weekend vs. weekday travel may be differentially associated with local environmental conditions.

Our study is limited by its cross-sectional design, and the possibility of self-selection at work in the five zones from which we collected data with a convenience sample. The high incidence of females (67% of the sample) and college graduates (84%) may be evidence of selection bias. The sample also is in the upper range in terms of meeting physical activity standards, whether judging from accelerometer or self-reported data [36]. Furthermore, it is possible that other scales--smaller or larger than the 1/2-mile buffer we used--would produce different results. Other sources of bias include defining each participant's neighborhood environment using a circle (Euclidean buffer) around the home location, rather than using the road network to identify accessible locations, and our reliance on outcome measures (step counts and trips) that were global (spatially non-specific), so we do not know how spatially coincident the steps and trips are with the environmental measures. For this reason, identifying activity within a neighborhood for which quantitative pedestrian environment measures are available may provide stronger relationships.

At the same time, the description of three different ways of quantifying each of several pedestrian features, and comparison of the results they yield in statistical models of walking behavior, provide useful methodological insights and ideas for further refining our techniques for measuring the environment. The limitations of analyzing data from a small sample such as this are compensated by the richness of the data we were able to collect. Beyond this exploratory examination at various aggregation techniques for environmental measures, future studies might benefit from other representations of segment proximity to a participant.

## Conclusion

This study found that associations between walking behavior (trips, steps) and pedestrian facilities are sensitive to measurement differences. More associations were discerned between pedestrian facilities and trips than between the same facilities and steps. Of the three aggregation techniques tested, the distance weighting shows the most promise, but also requires additional effort to calculate. Finally, single-exposure models (one pedestrian facility at a time) may provide information that is more useful to planners than the common indices or factors, which may obscure the relationship of walking with specific features relevant to planning and policy.

## Competing interests

The authors declare that they have no competing interests.

## Authors' contributions

ES conducted the statistical analysis and drafted the text of the paper. DR oversaw the participant data collection, developed initial hypotheses and methods, and reviewed all model results and drafts. GC generated environmental measures and performed data aggregation. KC conceived of and oversaw the audit data collection, oversaw participant data collection, and provided manuscript feedback. KE assisted with the data collection instruments and the development of hypotheses, and commented on manuscript drafts. All authors read and approved the final manuscript.
